# Green method for efficient PdNPs deposition on carbon carrier in the microreactor system

**DOI:** 10.1007/s11051-018-4337-9

**Published:** 2018-09-07

**Authors:** Magdalena Luty-Błocho, Marek Wojnicki, Grzegorz Włoch, Krzysztof Fitzner

**Affiliations:** Faculty of Non-Ferrous Metals, AGH University of Science and Technology, Al. A. Mickiewicza 30, 30-059 Krakow, Poland

**Keywords:** PdNP synthesis, Active carbon fibers, Catalyst, Microreactor, Continuous synthesis, Nanostructured catalyst

## Abstract

The synthesis of palladium nanoparticles and conditions of their deposition on active carbon fibers in the microreactor was described. All processes related with metal ion reduction, nucleation, and autocatalytic growth of particles as well as their deposition were carried out in the microreactor in only one cycle. Synthesis of palladium nanoparticles was carried out under different conditions, i.e., changing the initial concentration of metal ions and the reductant, at 40 °C. Depending on the conditions imposed, the nanoparticles of different size (hydrodynamic radius change from 12 to 37 nm) and shape (spherical, cube, pyramid) were obtained. It was also shown that flow conditions allow for much more efficient Pd deposition on active carbon fibers than the process carried out in the batch. It was observed that for concentrations of Pd(II) ions higher than 0.2 mM, the degree of fiber surface coverage increased significantly in comparison with the batch process.

**Electronic supplementary material:**

The online version of this article (10.1007/s11051-018-4337-9) contains supplementary material, which is available to authorized users.

## Introduction

Due to their unique physicochemical properties and depending on their morphology, palladium nanoparticles (PdNPs) may find an application in catalysis (Das et al. 2015; Gómez-Martínez et al. 2015; Lemo et al. 2006; Li et al. 2014; Magdesieva et al. 2014; Ncube et al. 2015; Reyes-Rios and García 2012; Shen et al. 2011), sensors (Chen et al. 2016; Choi et al. 2015; Cincotto et al. 2017; Gupta et al. 2014), fuel cell (Brandão et al. 2010; Ohara et al. 2009), and hydrogen storage (Dündar-Tekkaya and Yürüm 2016; Fang et al. 2015; Faye et al. 2017; Ma et al. 2015; Nair et al. 2015; Pang and Li 2016; Thanh et al. 2016; Viswanathan 2017).

Our attention was attracted by the process of catalysis, which uses 73.5% of the world’s palladium production (https://www.advantagefutures.com/outlook-2016/). It also seems that the demand for palladium will grow due to the rapid development of the industry. It is known that the best catalytic properties are associated with nanoparticles size, due to high surface to volume ratio. However, for many systems production, heterogeneous catalyst, even with excellent catalytic properties, is not enough. It is known that an equally important feature of catalysts is the ease of removing them from the gas or liquid-phase reaction mixture, as well as their ability to be reused. For the first purpose, catalyst supports like active carbon, Al_2_O_3_, TiO_2_, and SiO_2_ are applied. The choice of these materials consists in their inert behavior in various environments. The process of catalyst arrangement on a support can be achieved using precipitation and impregnation method (Munnik et al. 2015; State et al. 2017), conducted in a batch reactor. Precipitation techniques induce metal particle growth by supersaturation of the precursor solution, resulting in metal particle nucleation and growth. This can be done in conjunction with the formation of the support (coprecipitation) or on an existing support (precipitation deposition). Impregnation techniques bring the support into contact with a precursor solution. Low loadings of noble metals on a catalyst carrier are achieved by adsorption of the metal precursor by surface groups existing on the support, after which excess precursor is removed. Otherwise (higher loadings), the washing step is skipped and the support is dried directly so that whole amount of the precursor ends up on the support (impregnation and drying). Catalytic particles may also be formed in the colloidal route, in which metal precursor is reduced using reductant and the formed particles size and shape can be additionally controlled using stabilizers or ligands. After that, the formed particles are deposited on the support. The vapor deposition includes metal atoms, clusters, or organic substances in the gaseous phase, that selectively react with carrier surface groups (Munnik et al. 2015).

Such a process is multistage, takes some time, and requires further product purification. Thus, it is desirable to develop a relatively cheap and simple technique of particle production, which will eliminate these disadvantages and shorten the time of catalyst preparation. One of the ways to improve the process is its transfer from macroscale to microscale, which can be realized in microsystems (Ehrfeld et al. 2000). The term microsystem should be understood as a system composed of microreactors, mixers, microcapillaries, and pumps. Most advantages of the microreactor result from its small channel dimensions (high surface to volume ratio), which allow for fast reagents mixing, very rapid heat and mass transfer, controlled residence time of reagents in the microchannel realized by, e.g., flow rate and dimension of the channels, low reagent consumption, and a small amount of generated waste. Besides, the microsystem is flexible, because it allows for the connection of many reactors together, if a process requires a lot of reagents. It can also be applied to multistage processes, which require different conditions each. The microsystem allows the process intensification (Yao et al. 2015), and may find the application in such areas as biodiesel production (Madhawan et al. 2018), catalysis (Tanimu et al. 2017), green and sustainable synthesis, nanoparticle synthesis (Wojnicki et al. 2015; Zhao et al. 2011), and their deposition on the active carbon (Luty-Błocho et al. 2013) as well as metal particle synthesis in aqueous solution even at temperatures above 100 °C (Luty-Błocho et al. 2014).

In this work, we have explored the conditions necessary to obtain PdNPs in one-step continuous process, and their deposition on carbon fibers.

## Experiment

### Materials

#### Precursor of metallic palladium

The basic solution of Pd(II) ions was 0.1127 M of [PdCl_4_]^2¯^. The stock solution was prepared by dissolving of 14.8846 g Pd (pure 99.99; Mennica Państwowa, Poland) in an aqua regia (mixture of concentrated hydrochloric and nitric acid in volume ratio 3:1, POCH; Gliwice, Poland) repeated evaporation to dryness and dilution in deionized water repeated twice (Wojnicki et al. 2016).

#### Reductant

L-ascorbic acid (Sigma-Aldrich, Germany) in proper amount was dissolved to obtain the desired concentration of the reductant. All reagents used in experiments were pure grade.

#### Active carbon fibers as PdNP carrier

In order to prepare nanoparticle carrier, the commercially available carbon mate was applied. Activation of the carbon fiber surface was made according to the procedure described previously (Muszynski et al. 2008). For this purpose, 2.5 g of carbon fibers was added to the mixture of sulfuric acid (44 mL) and nitric acid (29 mL). Agitation of the mixture was carried out very carefully in a fume cupboard over 30 min, where potassium chlorate was added. Then, the mixture was stirred under a fume hood for 5 days until the stripping of “yellow gases” came from nitric dioxide. After this time, the mixture changed the color from yellow to blue and the obtained suspension was diluted with 2 l of deionized water and then filtered. The precipitate was additionally rinsed with 250 ml of 5% solution of hydrochloric acid. Finally, the carbon fibers were rinsed twice with deionized water to remove excess of HCl. The filtered carbon fibers were dried at 80 °C for approximately 12 h. In all experiments, 0.01 g of active carbon fibers was used.

### Sample analysis

#### Spectrophotometer

UV-Vis (Shimadzu, Japan), working in the wavelength range of 190–900 nm, was used to register the kinetics of the reaction and to analyze plasmon resonance, respectively. In order to measure the size and size distribution of the obtained particles, Nanozetasizer Nano-ZS (Malvern, UK) was used. The microstructure observations were performed using scanning electron microscopy (SEM; Hitachi U-70, Japan).

#### Sample preparation for spectrum analysis

The samples with the PdNPs were analyzed spectrophotometrically (plasmon) 10 min after collection of about 2 ml of the solution. The sample of colloidal palladium collected during experiments was examined twice (before and after passing the solution with PdNPs through the filter with ACF).

#### Sample preparation for dynamic light scattering analysis

Directly after plasmon registration, the solution with colloidal palladium was analyzed (size and size distribution) using dynamic light scattering (DLS) (Nanozetasizer Nano-ZS, Malvern, UK) method.

#### Sample preparation for microscope analysis

Immediately after collection of PdNPs for spectrum analysis, i.e., 1 min, after collecting the required volume for spectra and DLS analysis, the Pd/ACF was removed from the filter and dried at 50 °C for 4 h. After this time, the catalyst was cooled down, and was glued by Nafion to SEM sampler and dried again at room temperature for at least 12 h. The prepared sample was analyzed with the use of SEM microscope.

### Experimental setup

**Fig. 1:**
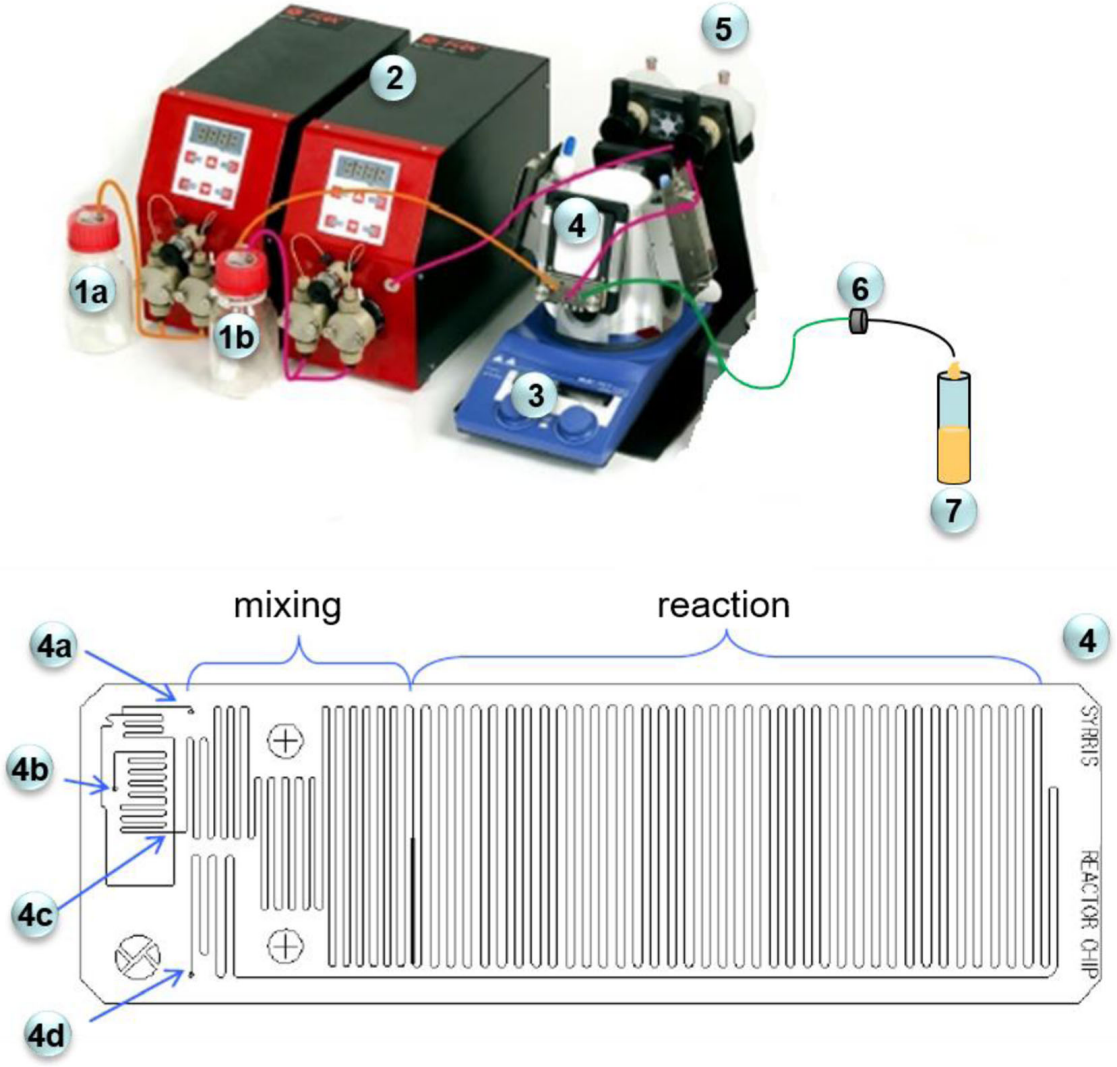
The scheme of microreactor system used for continuous PdNP synthesis and their deposition on active carbon fibers. 1a—a reservoir for the reducer, 1b—water, 2—a pump, 3—a thermostat, 4—a microchip, 4a—reductant input, 4b—metal precursor input, 4c—double T-mixing junction, 4d—fluid output, 5—a reservoir for Pd(II) ions, 6—filter with active carbon fibers, 7—sample

The palladium nanoparticle synthesis was conducted in the flow microreactor (Syrris, UK) with total volume 250 μl (mixing zone parameters: channel deep 250 μm, wide 300 μm; reacting zone: deep 250 μm, wide 400 μm, and length 2509 mm; Fig. 1). The microreactor was coupled with two syringe pumps operating at a flow rate from 0.001 to 10 ml/min, and a Teflon capillary that led the solution from the microreactor to the sampler. The microreactor contains two inputs: one for an aqueous solutions of L-ascorbic acid and another one for Pd(II) ions. Reagents are mixed in a micromixer and then directed to the reacting channel where the reduction reaction, the nucleation, and subsequent growth of palladium nanoparticles take place. The microreactor system is shown in Fig. 1.

As a result of the synthesis conducted in a microflow, the palladium nanoparticles were obtained and deposited on a carbon carrier. Synthesized nanoparticles were analyzed spectrophotometrically. The morphology and size distribution of synthesized materials were analyzed using DLS method and SEM.

### Experimental conditions

Synthesis of palladium nanoparticles was carried out under different conditions shown in Table 1.

**Table 1:** Experiment conditions for palladium nanoparticle synthesis in microreactor and PdNP deposition on ACF

Reagents concentration		Temperature, *T* (°C)	pH
*C*_0,Pd(II)_ (mM)	*C*_0,H2Asc_ (mM)		
An influence of initial concentration of metal precursor			
0.05	0.1	40	3.1
0.10	0.2		
0.20	0.4		
0.30	0.6		
0.40	0.8		
0.50	1.0		
An influence of initial concentration of reductant			
0.2	0.1		
	0.2		
	0.4	40	3.5
	0.6		
	0.8		

### Relation between kinetic study and flow parameters

The processes of reduction and particle deposition on carbon support in the flow require kinetic data which are necessary to flow parameter calculation. For this purpose, we used kinetic data given in our previous work. Based on that previous study (Wojnicki et al. 2016), the optimal resistance time *t*_0_ and total flow rate (i.e., 6.0 ml/min) in the microreactor were established (see Supplementary materials) from the obtained sigmoidal curves related to slow nucleation and fast autocatalytic growth. Constant temperature for nanoparticle synthesis in the flow was also selected (40 °C).

## Results and discussion

In order to optimize the size and the size distribution of PdNPs, two variables can be adjusted at constant temperature, i.e., the concentrations of Pd(II) ions and the reductant.

### The influence of initial concentration of metal precursor

**Fig. 2:**
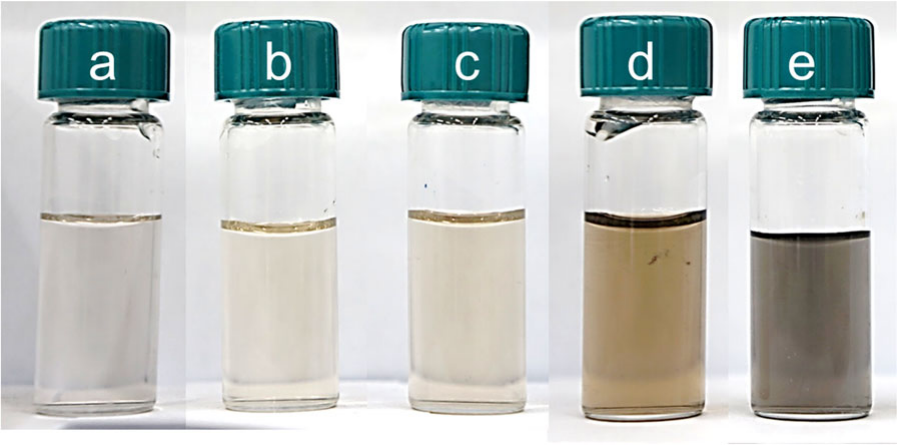
Colloidal palladium obtained in microreactor as a result of reduction reaction Pd(II) ions with L-ascorbic acid. Conditions: C_0, Pd(II)_ = 0.05–0.4 mM, C_0, AA_ = 0.1 (**a**)–0.8 mM (**f**), *T* = 40 °C, C_0,Cl¯_ = 0.0 M

The process of PdNP synthesis was carried out at different initial concentration of metal precursor in the range of 0.05–0.4 mM. To keep the constant excess of the reductant, the molar ratio of Pd(II) ions to ascorbic acid was maintained 1:2 (Table 2) in the experiment. As a result of the synthesis conducted in the microreactor, colloidal palladium having different color was obtained (Fig. 2a–e). The change of color from light yellow which is typical of Pd(II) ions into orange, brown, and gray suggests formation of metal on zero oxidation state.

**Table 2:** The values of the radius (**R**_**n**_**, R**_**i**_, calculated by the number (n) and the intensity (i), respectively) of palladium nanoparticles with standard deviation (σ)

C_0, Pd(IV)_ (mM)	C_0, AA_ (mM)	R_n_ ± σ (nm)	R_i_ ± σ (nm)
0.05	0.1	23.9 ± 7.9	57.3 ± 25.9
0.1	0.2	23.5 ± 7.7	54.5 ± 23.2
0.2	0.4	15.6 ± 4.1	29.0 ± 11.2
0.3	0.6	12.3 ± 3.4	24.4 ± 9.6
0.4	0.8	13.1 ± 3.7	25.0 ± 9.3

Conditions: C_0, Pd(II)_ = 0.05–0.4 mM, C_0, AA_ = 0.1–0.8 mM, *T* = 40 °C, *I* = 0.0 M, C_0, Cl¯_ = 0.0 M

**Fig. 3:**
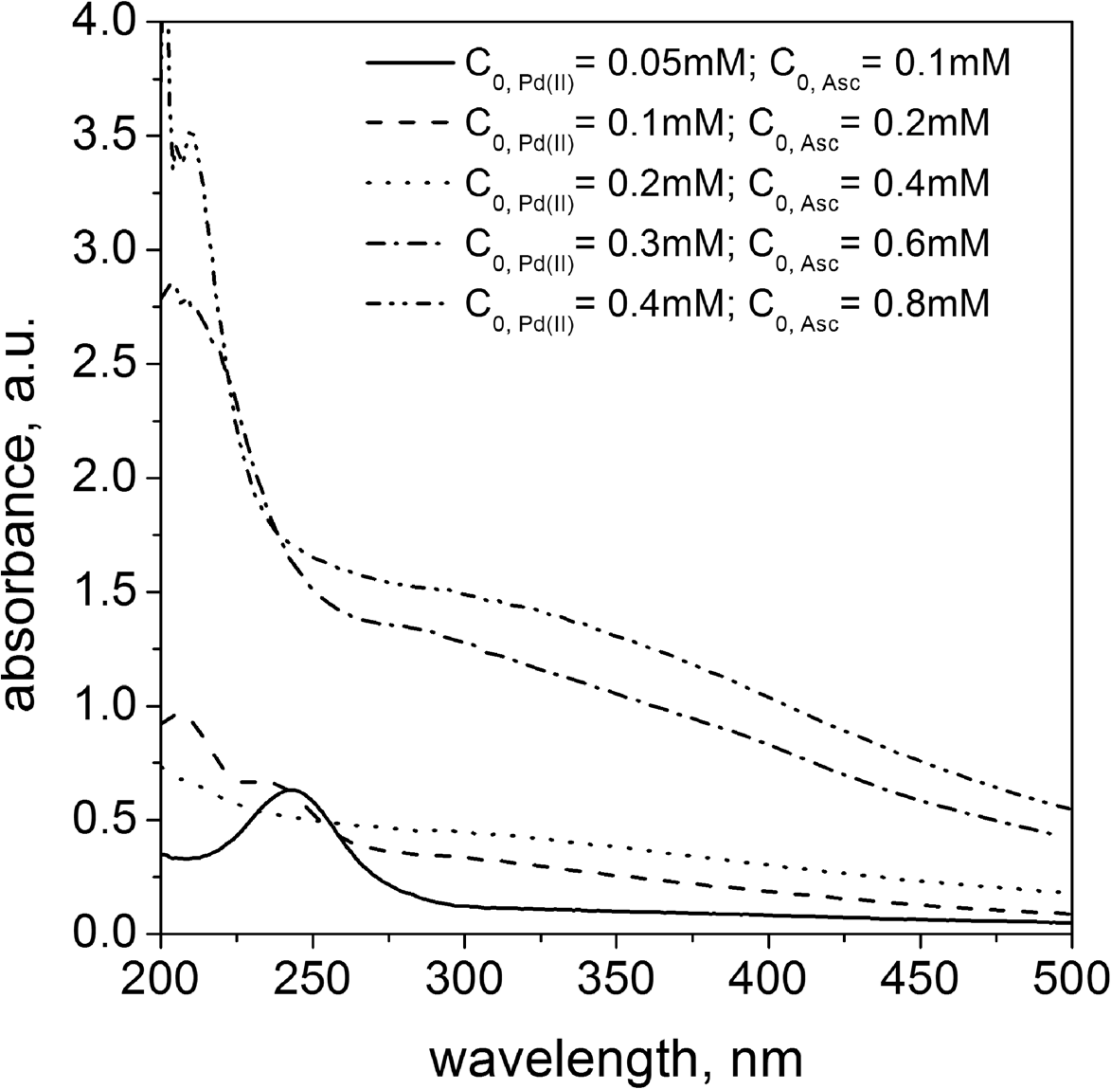
UV-Vis spectra of metallic phase obtained as a result of reduction reaction between Pd(II) ions and ascorbic acid conducted in microreactor at FR = 6.0 ml/min. Conditions: C_0, Pd(II)_ = 0.05–0.4 mM, C_0, AA_ = 0.1–0.8 mM, *T* = 40 °C, *I* = 0.0 M, C_0, Cl¯_ = 0.0 M

The obtained colloids were analyzed spectrophotometrically and the registered spectra are shown in Fig. 3. As it was expected, the highest level of turbidity was obtained for the highest amount of metal precursor. As a result of reaction between reagents with their smallest concentration (i.e., 0.05 mM Pd(II) and 0.1 mM ascorbic acid), a peak at about 250 nm was registered. Taking into account that reduction reaction between palladium(II) ions and ascorbic acid requires two donors, the observed peak can be associated with an unreacted reductant. For higher reductant concentration, the increase of absorbance level in the range of 280–500 nm was observed. In accordance with the literature, the plasmon resonance can be observed for palladium particles bigger in diameter than 40 nm (Sugawa et al. 2015). However, in practice, we do not observe any spectrum with visible maximum, which can be explained by unreacted subtract and/or product spectrum overlap.

The presence of nanoparticles was confirmed by the use of DLS method, and the obtained values of the radius are gathered in Table 2.

**Fig. 4:**
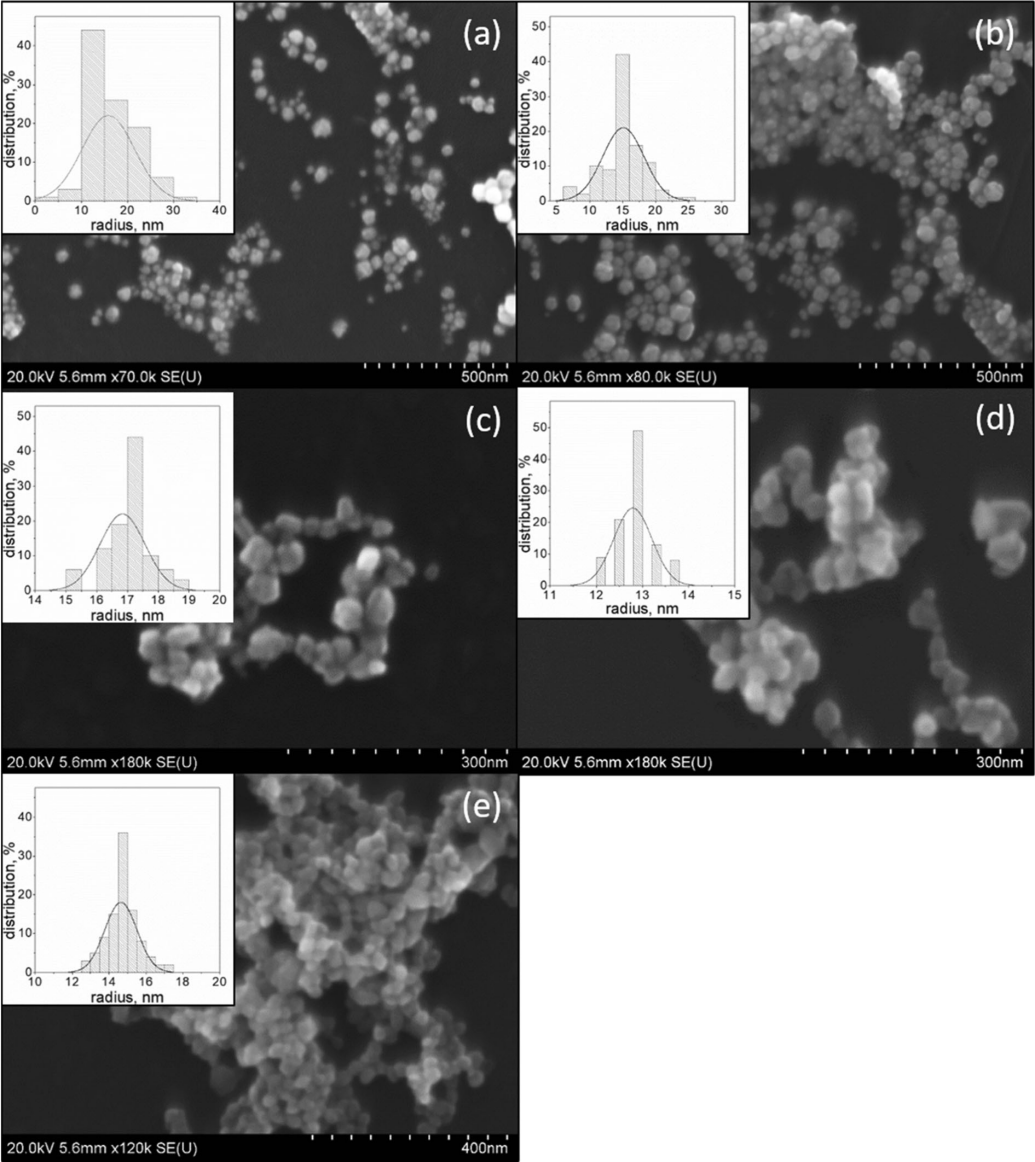
PdNPs obtained in results of reduction reaction Pd(II) chloride complex ions with ascorbic acid and their size distribution. Conditions: FR = 6.0 mL/min, *T* = 40 °C, *I* = 0.0 M, C_0, Cl¯_ = 0.0 M. **a** C_0, Pd(II)_ = 0.05 mM, C_0, AA_ = 0.1 mM. **b** C_0, Pd(II)_ = 0.1 mM, C_0, AA_ = 0.2 mM. **c** C_0, Pd(II)_ = 0.2 mM, C_0, AA_ = 0.4 mM. **d** C_0, Pd(II)_ = 0.3 mM, C_0, AA_ = 0.6 mM. **e** C_0, Pd(II)_ = 0.4 mM, C_0, AA_ = 0.8 mM

It was found that with an increasing amount of palladium(II) ions, the size of particles is decreasing. It can also be seen that polydispersity also decreases. In order to confirm the obtained results, colloidal palladium was examined with SEM microscopy (Fig. 4).

SEM analysis showed that the size of obtained palladium nanoparticles slightly decreases with increasing initial concentration of Pd(II) ions in the reacting solution. The size distribution also decreases. The obtained palladium nanoparticles are irregular in shape and usually form spherical clusters. Some aggregates were also formed.

### The influence of initial concentration of reductant

**Fig. 5:**
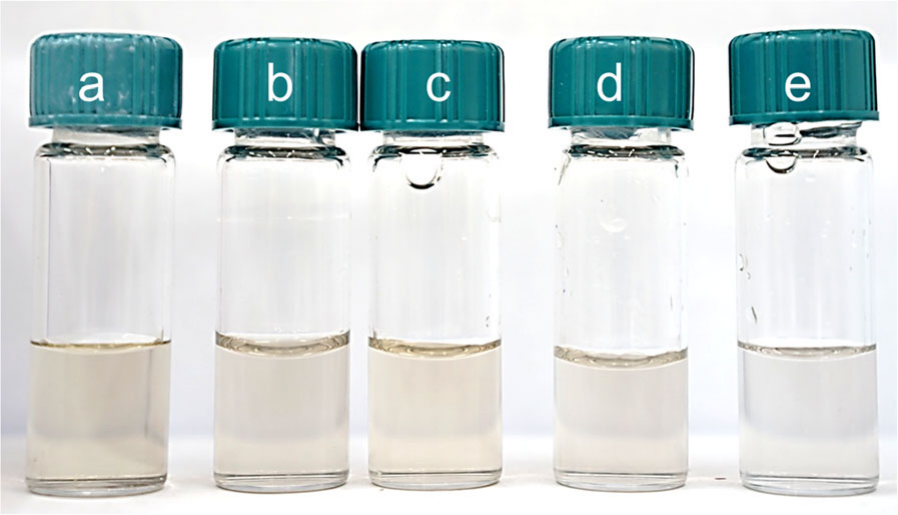
Colloidal palladium obtained in microreactor as a result of reduction reaction of Pd(II) ions with L-ascorbic acid. Conditions: C_0, Pd(II)_ = 0.2 mM, C_0, AA_ = 0.1 (**a**)–0.8 mM (**f**), *T* = 40 °C, *I* = 0.0 M, C_0, Cl¯_ = 0.0 M

As a result of mixing 0.2 mM solution of the chloride palladium with different amounts of ascorbic acid in the flow microreactor, colloids with a “light brown” color were obtained (Fig. 5a–e). The change in color indicates that a solid phase appears in the analyzed solutions.

**Fig. 6:**
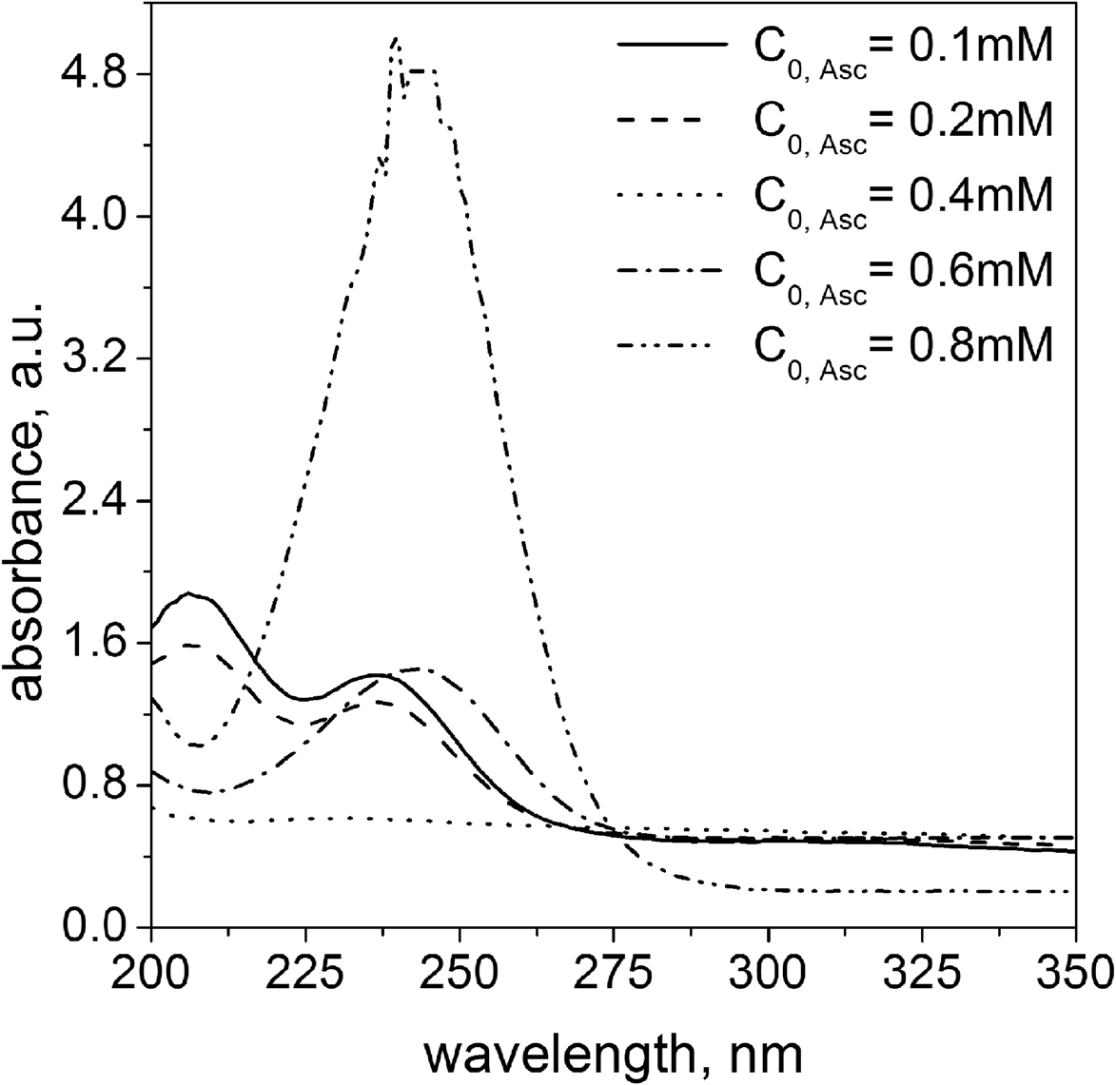
UV-Vis spectra of metallic phase obtained as a result of reduction reaction between Pd(II) ions with ascorbic acid conducted in microreactor at FR = 6.0 mL/min. Conditions: C_0, Pd(II)_ = 0.2 mM, C_0, AA_ = 0.1–0.8 mM, *T* = 40 °C, *I* = 0.0 M, C_0, Cl¯_ = 0.0 M

It was confirmed when solutions containing palladium were analyzed spectrophotometrically. The obtained results are shown in Fig. 6. In all cases, an increase in the absorbance in the visible light was observed, indicating the appearance of the metallic phase. The presence of palladium nanoparticles was confirmed by dynamic light scattering experiment, and the obtained values of hydrodynamic radius are summarized in Table 3. For smaller amount of ascorbic acid, i.e., 0.1 and 0.2 mM, unreacted Pd(II) ions were identified, since on registered spectrum, two peaks are visible, which are characteristic of palladium complex (λ_max,1_ = 207 nm and λ_max,2_ = 236 nm). For higher amount of reductant, unreacted ascorbic acid was identified, i.e., the characteristic peak was observed at about 250 nm.

**Table 3:** The values of radius (**R**_**n**_**, R**_**i**_, calculated by the number (n) and the intensity (i), respectively) of palladium nanoparticles with standard deviation (σ)

C_0, AA_ (mM)	R_n_ ± σ (nm)	R_i_ ± σ (nm)	Color of the solution
0.1	24.6 ± 6.4	41.9 ± 14.2	Pale brown
0.2	31.0 ± 8.6	50.2 ± 15.8	
0.4	20.5 ± 6.0	45.9 ± 20.5	
0.6	25.5 ± 7.6	51.2 ± 20.3	
0.8	37.1 ± 14.4	70.1 ± 27.2	

Conditions: C_0, Pd(II)_ = 0.2 mM, C_0, AA_ = 0.1–0.8 mM, *T* = 40 °C, *I* = 0.0 M, C_0, Cl¯_ = 0.0 M

**Fig. 7:**
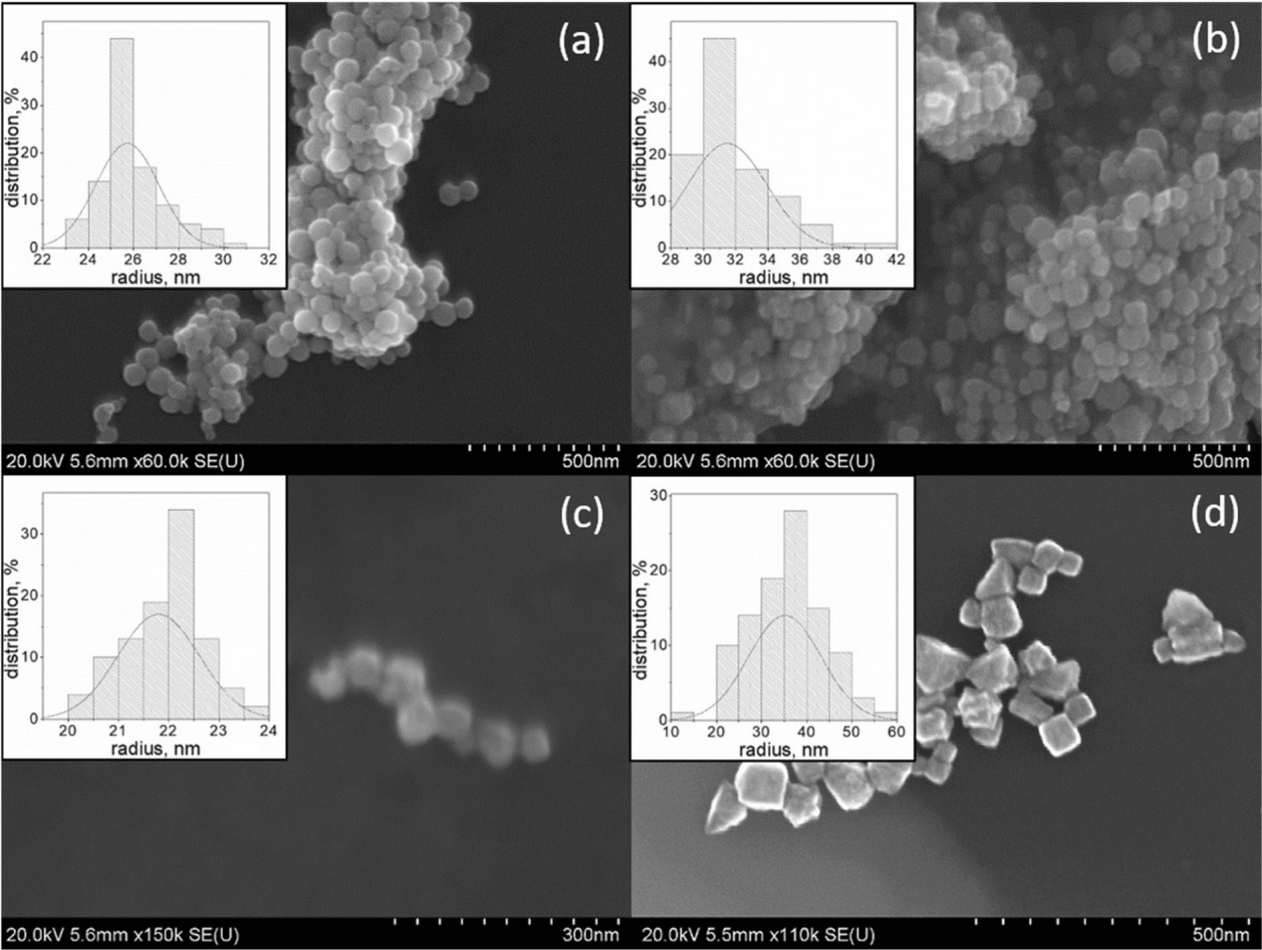
PdNPs obtained as a result of reduction reaction Pd(II) chloride complex ions with ascorbic acid and their size distribution. Conditions: *T* = 40 °C, *I* = 0.0 M, C_0, Cl¯_ = 0.0 M, C_0, Pd(II)_ = 0.2 mM. **a** C_0, AA_ = 0.1 mM. **b** C_0, AA_ = 0.2 mM. **c** C_0, AA_ = 0.4 mM. **d** C_0, AA_ = 0.8 mM

Basing on the obtained hydrodynamic radius (Table 3), it is difficult to find the influence of the concentration of the reductant on the size of palladium nanoparticles. The radius values (*R*_*n*_) ranged from 20.5 ± 6.0 to 37.1 ± 14.4 nm. In addition, the synthesized palladium nanoparticles were analyzed using SEM, and the obtained results are shown in Fig. 7a–d. For the lowest initial concentration of L-ascorbic acid, spherical particles (Fig. 7a) with a diameter of about 50 nm were obtained.

For higher concentrations of the reducer, i.e., 0.4 and 0.8 mM, palladium nanoparticles of different shapes like sphere, tetrahedron, and cube are obtained (Fig. 7c, d). These shapes suggest that the nanoparticle growth mechanism and their appearance can be controlled by a reducer, which confirms our previous findings (Luty-Błocho et al. 2018).

### PdNP deposition on ACF

#### Deposition in microflow

Having determined the influence of Pd(II) and reductant concentration on the size and shape, we used this knowledge to produce hybrid material. It can be done by mixing metal precursor with reductant in volume ratio 1:1 in a microreactor chip (part 4 in Fig. 1). In the first stage, reagents are introduced to the separate microchannels in a microchip. The stream (4a; Fig. 1) consisting of reductant is split into two parts, which are directed to the *T*-shaped micromixer (4c; Fig. 1). In this place, two streams with reductant are introduced to the stream with metal ions and the mixing process starts. After that, the reduction reaction between Pd(II) ions and L-ascorbic acid takes place followed by steps like nucleation and autocatalytic grow of palladium nanoparticles. As a result of this process, at the end of the reactor, palladium nanoparticles are formed.

**Fig. 8:**
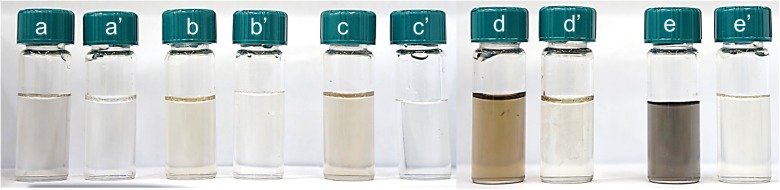
Colors obtained solutions containing palladium nanoparticles resulting from the reduction of the chloride complex of Pd (II) with L-ascorbic. Conditions: C_0, Pd(II)_ = 0.05–0.4 mM, C_0, AA_ = 0.1 (**a**)–0.8 mM (**e′**), *T* = 40 °C, *I* = 0.0 M, C_0, Cl¯_ = 0.0 M. **a**–**e** denote sample before solution passing through filter with ACF, and **a′**–**e′** after solution passing through filter

After synthesis, the palladium particles are directed to the microcapillary with the filter in which carbon carrier was closed (Fig. 1). The obtained solutions with PdNPs were analyzed twice, first before and then after passing through the filter with carbon fibers. The results of these experiments (Fig. 8) show that the solutions after passing through the filter “discolored.” This demonstrates that palladium nanoparticles are adsorbed on the surface of the active carbon fibers.

**Fig. 9:**
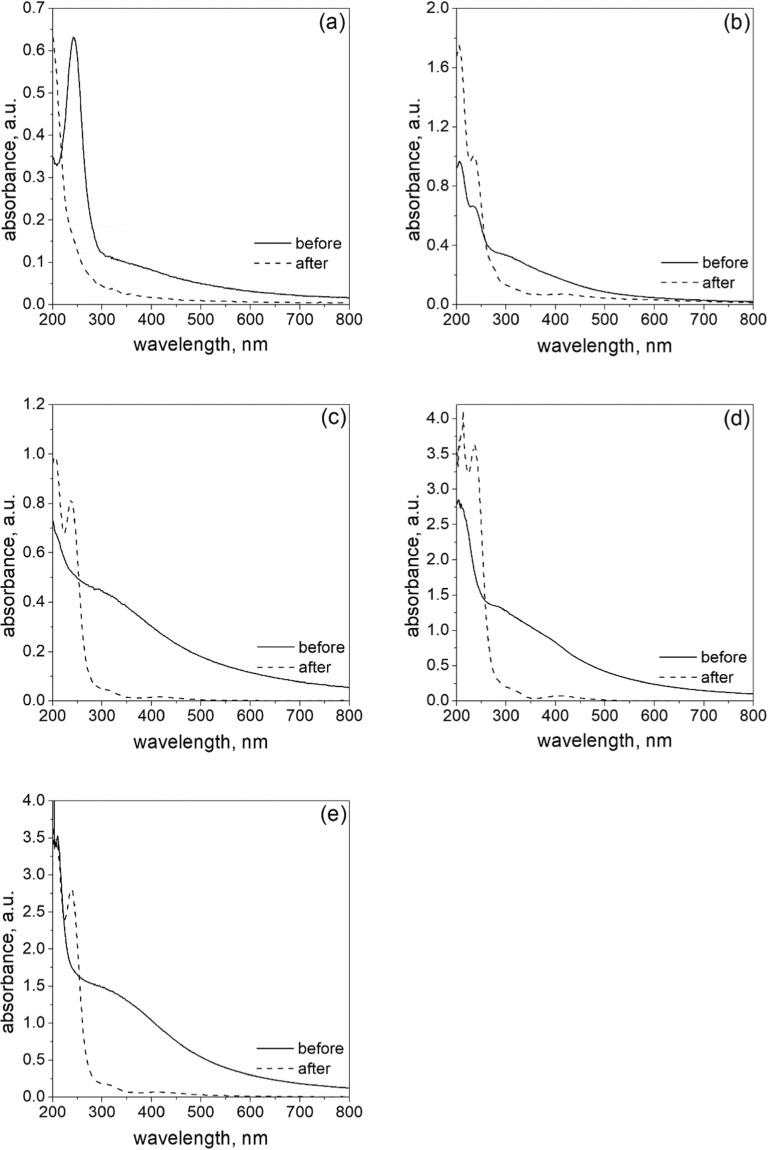
UV-Vis spectrum colloidal phase of palladium obtained as a result of reduction reaction of Pd(II) ions with L-ascorbic acid. Conditions: FR = 6.0 mL/min, *T* = 40 °C, *I* = 0.0 M, C_0, Cl¯_ = 0.0 M, C_0, Pd(II)_ = 0.05 mM, C_0, AA_ = 0.1 mM (**a**). C_0, Pd(II)_ = 0.1 mM, C_0, AA_ = 0.2 mM (**b**). C_0, Pd(II)_ = 0.2 mM, C_0, AA_ = 0.4 mM (**c**). C_0, Pd(II)_ = 0.3 mM, C_0, AA_ = 0.6 mM (**d**). C_0, Pd(II)_ = 0.4 mM, C_0, AA_ = 0.8 mM (**e**)

The obtained colloids were analyzed sepctrophotometrically and the obtained results are shown in Fig. 8a–e. For each colloid, characteristic spectra in the visible range were registered (Fig. 9). It was observed that spectral intensity related to the solution after passing it through the filter with active carbon fibers decreases. The obtained results prove that palladium nanoparticles have been adsorbed on the ACF surface.

However, they also indicate that active carbon fibers may adsorb ascorbic acid (see Fig. 9a), which was confirmed by a separate test (see, Supplementary materials, Fig. S1). Moreover, for higher amount of Pd(II) ions in the solution, rebuilding of the spectrum band was registered with maximum at 207 and 236 nm after passing to the filter. The registered spectrum indicates the presence of Pd(II) ions in the waste solution, and suggests that following reactions take place:

1 $$ Pd+\frac{1}{2}{O}_2\leftrightarrow Pd O $$

2 $$ PdO+ HCl\leftrightarrow PdC{l}_2+{H}_2O $$

which yields overall reaction

3 $$ Pd+\frac{1}{2}{O}_2+2 HCl\leftrightarrow Pd C{l}_2+{H}_2O $$

For this reaction ΔG_T_° = −269 kJ and the obtained negative value of Gibbs free energy means that this process (i.e., reaction between metallic palladium and oxygen dissolved in the solution) is spontaneous.

Samples of colloidal palladium used in experiments were also analyzed using DLS method, and the results are summarized in Table 4.

**Table 4:** The values of radius (**R**_**n**_**, R**_**i**_, calculated by number (n) and intensity (i), respectively) of palladium nanoparticles with standard deviation (σ)

C_0, Pd(IV)_ (mM)	C_0, AA_ (mM)	Before *R*_*n*_ ± σ (nm)	After *R*_*n*_ ± σ (nm)	Before *R*_*i*_ ± σ (nm)	After *R*_*i*_ ± σ (nm)	Color
0.05	0.1	23.9 ± 7.9	29.0 ± 8.0	57.3 ± 25.9	49.0 ± 16.3	Brown
0.1	0.2	23.5 ± 7.7	18.2 ± 4.8	54.5 ± 23.2	31.4 ± 10.4	
0.2	0.4	15.6 ± 4.1	122 ± 22	29.0 ± 11.2	124 ± 18	
0.3	0.6	12.3 ± 3.4	20.5 ± 5.9	24.5 ± 9.6	42.1 ± 16.6	
0.4	0.8	13.1 ± 3.7	16.1 ± 5.0	25.0 ± 9.3	44.2 ± 22.8	

Conditions: C_0, Pd(II)_ = 0.05–0.4 mM, C_0, AA_ = 0.1–0.8 mM, *T* = 40 °C, *I* = 0.0 M, C_0, Cl¯_ = 0.0 M

**Fig. 10:**
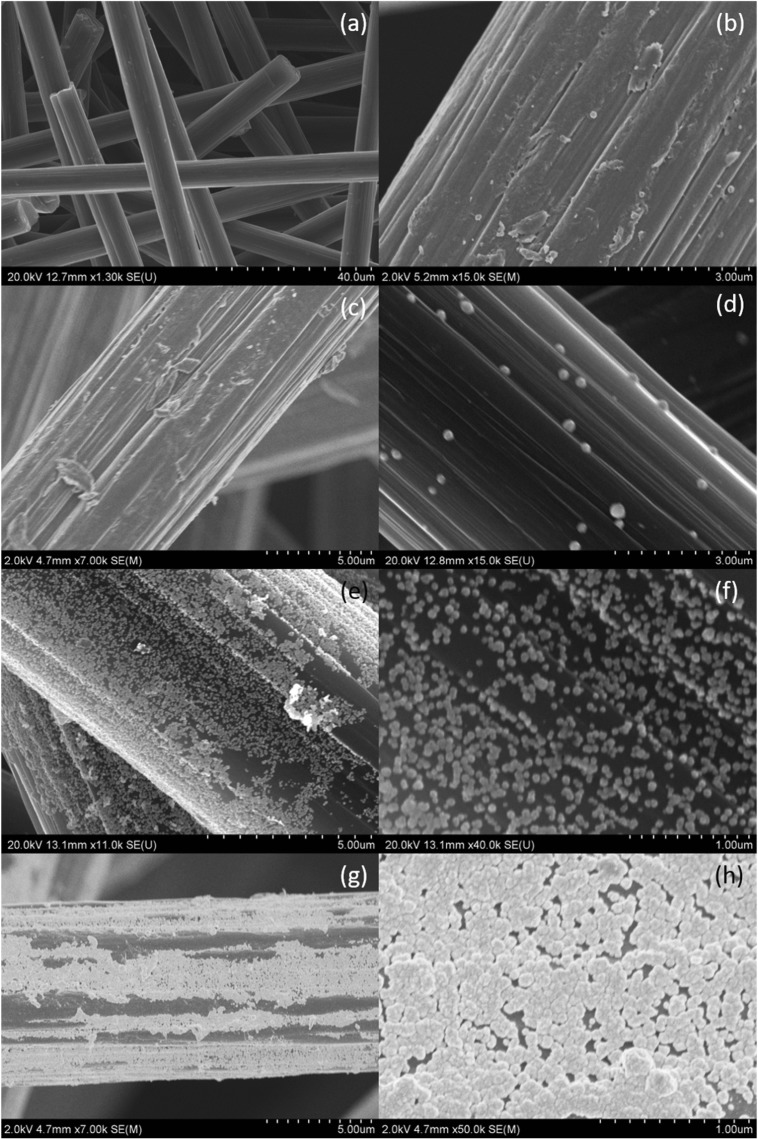
Pd/ACF catalyst obtained as a result of nanoparticle synthesis in microreactor and their deposition on active carbon fibers. Conditions: FR = 6.0 mL/min, *T* = 40 °C, *I* = 0.0 M, C_0, Cl¯_ = 0.0 M. **a** Active carbon fibers. **b** C_0, Pd(II)_ = 0.05 mM, C_0, AA_ = 0.1 mM. **c** C_0, Pd(II)_ = 0.1 mM, C_0, AA_ = 0.2 mM. **d** C_0, Pd(II)_ = 0.2 mM, C_0, AA_ = 0.4 mM. **e**, **f** C_0, Pd(II)_ = 0.3 mM, C_0, AA_ = 0.6 mM. **g**, **h** C_0, Pd(II)_ = 0.4 mM, C_0, AA_ = 0.8 mM

The values of the obtained hydrodynamic radius indicate that palladium nanoparticles are present in solutions before as well as after passing them through the filter with activated carbon fibers. This means that not all of the particles have been deposited on the ACF. Hydrodynamic radius values indicate that the size of the deposited particles should range from 12.3 ± 3.4 to 23.9 ± 7.9 nm (Table 4). Microscopic analyses were performed to verify the efficiency of the particle deposition process on activated carbon fiber surface. The results are presented in Fig. 10a–h.

It was found that the palladium nanoparticle deposition on activated carbon fibers (Fig. 10a) is not effective (Fig. 10b, c) for the lowest palladium(II) precursor concentrations, i.e., 0.05 and 0.1 mM. Too small amount of introduced ions makes the amount of synthesized nanoparticles small, and therefore, the degree of fibers coverage is negligible. For higher concentrations of Pd(II) ions, i.e., above 0.2 mM, the degree of fiber surface coverage increased significantly (Fig. 10d–h). The highest degree of carbon fiber coverage was obtained for the highest values of metal ion concentrations (Fig. 10e–h). It has also been observed that palladium nanoparticles (Fig. 10e, g) are separated from the fiber at some locations. For the highest concentration of the initial palladium precursor, it was noted that the particles “stick together” to form a nanometric layer (Fig. 10h).

#### Deposition in a batch reactor

**Fig. 11:**
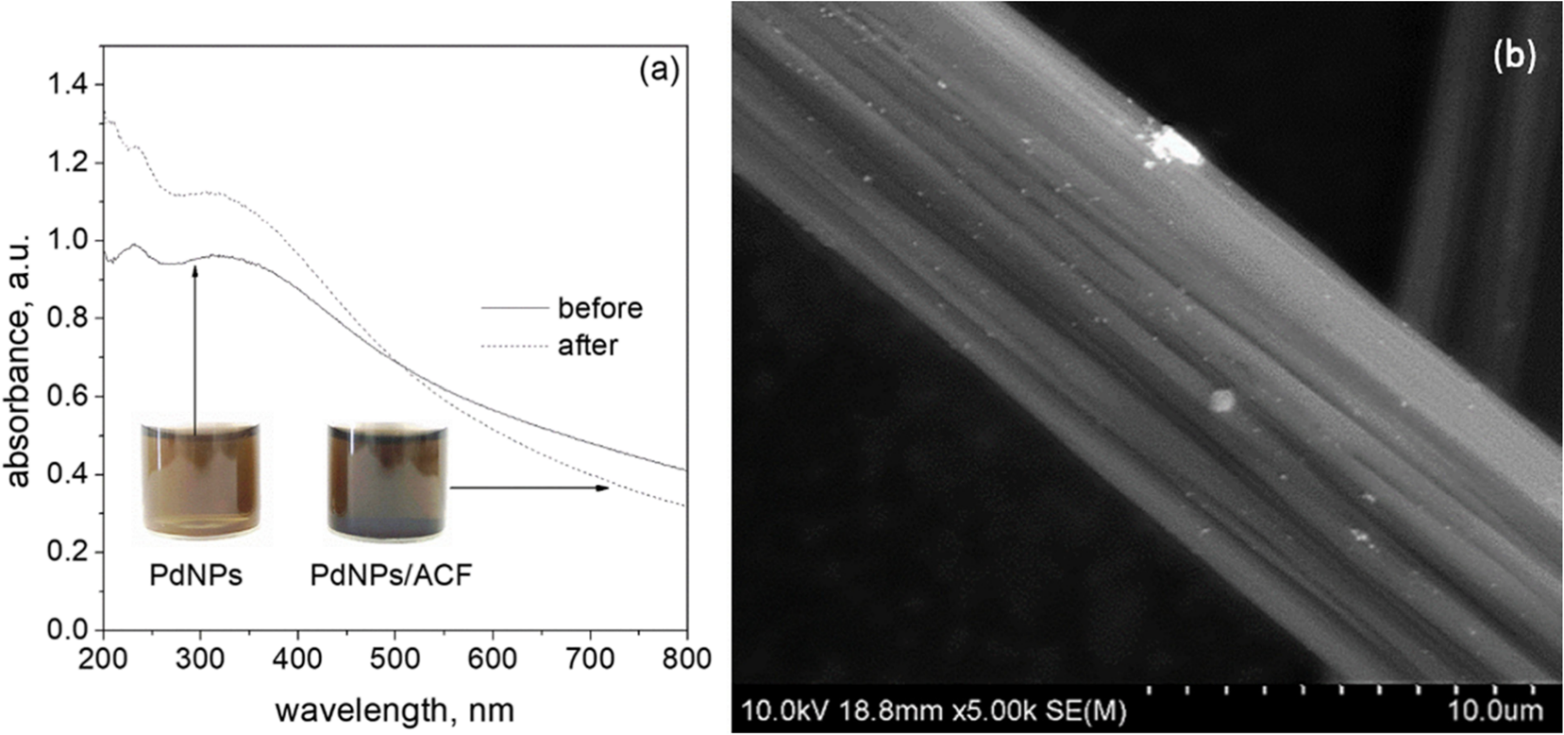
UV-Vis spectrum of the colloidal phase of palladium obtained as a result of reduction reaction of Pd(II) ions with L-ascorbic acid in a batch reactor. Conditions: *T* = 40 °C, *I* = 0.0 M, C_0, Pd(II)_ = 0.3 mM, C_0, AA_ = 0.6 mM (**a**). Pd/ACF obtained as a result of nanoparticles synthesis in a batch reactor and their deposition on active carbon fibers. Conditions: T = 40 °C, I = 0.0 M, C_0, Cl¯_ = 0.0 M, C_0, Pd(II)_ = 0.3 mM, C_0, AA_ = 0.6 mM (**b**)

In order to prove the advantage of a microreactor, the process of palladium nanoparticle deposition on ACF was also carried out in a batch reactor. This process for PdNP synthesis was conducted for selected conditions, i.e., 0.3 mM of Pd(II) ions and 0.6 mM of ascorbic acid at 40 °C, which were found to be optimal for the synthesis in a microreactor. Solutions of metal precursor and reductant were heated up to 40 °C, and then, they were simultaneously mixed in volumetric ratio 1:1. After few seconds, the yellow color of the solution (coming from Pd(II) ions) turned into brown. This color change and registered spectrum (Fig. 11a) confirm that nanoparticles are formed. This colloidal palladium was added to the active carbon fibers (0.01 g) and mixed. The process of impregnation was carried out for 1 h. Next, the active carbon fibers were separated from the colloidal palladium and dried at 50 °C for 4 h. The colloidal palladium before and after impregnation process was examined using spectrophotometric and DLS methods. In addition, active carbon fibers after impregnation stage were analyzed using SEM. The result of this analysis is shown in Fig. 11b.

As it can be seen (Fig. 11a), the spectrum for colloidal palladium changed after impregnation step. It suggests that either some palladium particles can be adsorbed on ACF or some aggregates are formed. Similarly to the synthesis carried out in a microreactor, the reappearance of the spectrum band was registered after impregnation stage with maximum at 207 and 236 nm. It was also observed that during impregnation of ACF, the solution changed color to darker one (Fig. 11a), and this result is opposite to the effect observed in a microreactor (Fig. 8d, d′). It suggests that the PdNP deposition on ACF carried out in a batch reactor is ineffective even after much longer time than it took in a microreactor. Hydrodynamic radius indicates that the size of particles obtained in a batch route before impregnation equals 8.4 ± 1.8 nm (95.5%) and 22.8 ± 9.8 (4.5%) (values calculated by number). After impregnation step, the size of obtained particles is equal to 21.1 ± 6.6 (100%). It suggests that during 1 h, the size of particles in the solution enlarged. Microscopic analysis was performed to verify the efficiency of the particle deposition process on activated carbon fiber surface. The result is presented in Fig. 11b. It can be seen that the amount of deposited particles on the ACF is much lower than in case of the process conducted in a microreactor (Fig. 10f).

## Conclusions

As a result of Pd(II) reduction with L-ascorbic acid and nucleation and growth of nanoparticles in a flow microreactor, it was possible to obtain palladium nanoparticles of different sizes, size distribution, and shapes in one cycle. On the one hand, no significant effect of the reducer concentration on the size of the resulting particles was observed. On the other hand, it has been observed that as the concentration of L-ascorbic acid increases, the shape of the resulting palladium nanoparticles is more diverse.

It was found that at constant temperature of 40 °C and the total flow rate of the reactants of 6.0 ml/min, palladium nanoparticles were obtained, which are similar in irregular shape and size in the range from 10 to 50 nm. Microscopic observations revealed the presence of aggregates (Fig. 10g, h) for higher values of concentration of Pd(II) ions.

The use of the flow allowed for the simultaneous synthesis of particles and their immediate deposition on carbon fibers in one step. Depending on the initial reactant concentrations, different levels of carbon fiber surface coverage were obtained. The best coverage and particles distribution of Pd deposited on ACF were obtained for 0.3 mM solution of metal precursor and 0.6 mM solution of reductant (Fig. 10e, f).The radius of the deposited palladium nanoparticles ranged from 25 to 30 nm. The experiment performed in the batch reactor carried out under optimal conditions speaks in favor of the process conducted in the microreactor. We showed that under optimal conditions, it is hardly possible to deposit PdNPs on ACF in the batch (Fig. 11b).

In general, application of microreactors has an advantage over batch reactor. Small size and high surface to volume ratio of the space for the reaction enable precise temperature control and high efficiency of heterogeneous mass transfer. Moreover, the possibility of combining reactors in parallel may enhance total production capacity over time. This technique can contribute to green sustainable chemical synthesis by decreasing energy consumption and reagents and by production of a small amount of waste. An additional advantage is the intensification of the process. The change in the construction of the microreactor may income the flow rates and to shorten residence time significant (Zhang et al. 2018).

## Electronic supplementary material

ESM 1 (DOCX 123 kb)

## References

[CR1] Brandão L, Rodrigues J, Madeira LM, Mendes A (2010). Methanol crossover reduction by Nafion modification with palladium composite nanoparticles: application to direct methanol fuel cells. Int J Hydrog Energy.

[CR2] Chen X, Li G, Zhang G, Hou K, Pan H, Du M (2016). Self-assembly of palladium nanoparticles on functional TiO_2_ nanotubes for a nonenzymatic glucose sensor. Mater Sci Eng C.

[CR3] Choi B, Ahn JH, Lee J, Yoon J, Lee J, Jeon M, Kim DM, Kim DH, Park I, Choi SJ (2015). A bottom-gate silicon nanowire field-effect transistor with functionalized palladium nanoparticles for hydrogen gas sensors. Solid State Electron.

[CR4] Cincotto FH, Golinelli DLC, Machado SAS, Moraes FC (2017). Electrochemical sensor based on reduced graphene oxide modified with palladium nanoparticles for determination of desipramine in urine samples. Sensors Actuators B Chem.

[CR5] Das RS, Singh B, Mandal A, Banerjee R, Mukhopadhyay S (2015). Kinetics of palladium nano-particles catalyzed reduction of methylene green by hydrazine: role of induction period in determining mechanistic pathway. Inorg Chim Acta.

[CR6] Dündar-Tekkaya E, Yürüm Y (2016). Synthesis of palladium incorporated MCM-41 via microwave irradiation and investigation of its hydrogen storage properties. Int J Hydrog Energy.

[CR7] Ehrfeld W, Hessel V, Löwe H (2000). Microreactors : new technology for modern chemistry.

[CR8] Fang J, Levchenko I, Lu X, Mariotti D, Ostrikov K (2015). Hierarchical bi-dimensional alumina/palladium nanowire nano-architectures for hydrogen detection, storage and controlled release. Int J Hydrog Energy.

[CR9] Faye O, Szpunar JA, Szpunar B, Beye AC (2017). Hydrogen adsorption and storage on palladium – functionalized graphene with NH-dopant: a first principles calculation. Appl Surf Sci.

[CR10] Gómez-Martínez M, Buxaderas E, Pastor IM, Alonso DA (2015). Palladium nanoparticles supported on graphene and reduced graphene oxide as efficient recyclable catalyst for the Suzuki–Miyaura reaction of potassium aryltrifluoroborates. J Mol Catal A Chem.

[CR11] Gupta D, Dutta D, Kumar M, Barman PB, Sarkar CK, Basu S, Hazra SK (2014). A low temperature hydrogen sensor based on palladium nanoparticles. Sensors Actuators B Chem.

[CR12] Lemo J, Heuzé K, Astruc D (2006). Synthesis and catalytic activity of DAB-dendrimer encapsulated Pd nanoparticles for the Suzuki coupling reaction. Inorg Chim Acta.

[CR13] Li Y, Dai Y, Yang Z, Li T (2014). Controllable synthesis of palladium nanoparticles and their catalytic abilities in heck and Suzuki reactions. Inorg Chim Acta.

[CR14] Luty-Błocho M, Wojnicki M, Pacławski K, Fitzner K (2013). The synthesis of platinum nanoparticles and their deposition on the active carbon fibers in one microreactor cycle. Chem Eng J.

[CR15] Luty-Błocho M, Wojnicki M, Grzonka J, Kurzydłowski KJ (2014). The synthesis of stable platinum nanoparticles in the microreactor. Arch Metall Mater.

[CR16] Luty-Błocho M, Wojnicki M, Grzonka J, Kurzydłowski K, Fitzner K (2018). Linking the gold nanoparticles formation kinetics with their morphology. In J Chem Kinet.

[CR17] Ma L, Zhang J-M, Xu K-W, Ji V (2015). Hydrogen adsorption and storage on palladium-decorated graphene with boron dopants and vacancy defects: a first-principles study. Physica E Low Dimens Syst Nanostruct.

[CR18] Madhawan A, Arora A, Das J, Kuila A, Sharma V (2018). Microreactor technology for biodiesel production: a review. Biomass Convers Biorefin.

[CR19] Magdesieva TV, Nikitin OM, Zolotukhina EV, Vorotyntsev MA (2014). Palladium nanoparticles–polypyrrole composite as an efficient catalyst for cyanation of aryl halides. Electrochim Acta.

[CR20] Munnik P, de Jongh PE, de Jong KP (2015). Recent developments in the synthesis of supported catalysts. Chem Rev.

[CR21] Muszynski R, Seger B, Kamat PV (2008). Decorating graphene sheets with gold nanoparticles. J Phys Chem C.

[CR22] Nair AAS, Sundara R, Anitha N (2015). Hydrogen storage performance of palladium nanoparticles decorated graphitic carbon nitride. Int J Hydrog Energy.

[CR23] Ncube P, Bingwa N, Baloyi H, Meijboom R (2015). Catalytic activity of palladium and gold dendrimer-encapsulated nanoparticles for methylene blue reduction: a kinetic analysis. Appl Catal A Gen.

[CR24] Ohara S, Hatakeyama Y, Umetsu M, Sato K, Naka T, Adschiri T (2009). Palladium–polyelectrolyte hybrid nanoparticles for hydrogen sensor in fuel cells. J Power Sources.

[CR25] Pang Y, Li Q (2016). A review on kinetic models and corresponding analysis methods for hydrogen storage materials. Int J Hydrog Energy.

[CR26] Reyes-Rios G, García JJ (2012). Alkylation of amines with alcohols catalyzed by palladium nanoparticles. Inorg Chim Acta.

[CR27] Shen C, Wang YJ, Xu JH, Lu YC, Luo GS (2011). Preparation and the hydrogenation performance of a novel catalyst-Pd nanoparticles loaded on glass beads with an egg–shell structure. Chem Eng J.

[CR28] State R, Scurtu M, Miyazaki A, Papa F, Atkinson I, Munteanu C, Balint I (2017). Influence of metal-support interaction on nitrate hydrogenation over Rh and Rh-cu nanoparticles dispersed on Al2O3 and TiO2 supports. Arab J Chem.

[CR29] Sugawa K, Tahara H, Yamashita A, Otsuki J, Sagara T, Harumoto T, Yanagida S (2015). Refractive index susceptibility of the plasmonic palladium nanoparticle: potential as the third plasmonic sensing material. ACS Nano.

[CR30] Tanimu A, Jaenicke S, Alhooshani K (2017). Heterogeneous catalysis in continuous flow microreactors: a review of methods and applications. Chem Eng J.

[CR31] Thanh TD, Balamurugan J, Lee SH, Kim NH, Lee JH (2016). Novel porous gold-palladium nanoalloy network-supported graphene as an advanced catalyst for non-enzymatic hydrogen peroxide sensing. Biosens Bioelectron.

[CR32] Viswanathan Balasubramanian (2017). Hydrogen Storage. Energy Sources.

[CR33] Wojnicki M, Luty-Błocho M, Mech K, Grzonka J, Fitzner K, Kurzydłowski K (2015). Catalytic properties of platinum nanoparticles obtained in a single step simultaneous reduction of Pt(IV) ions and graphene oxide. J Flow Chem.

[CR34] Wojnicki M, Fitzner K, Luty-Błocho M (2016). Kinetic studies of nucleation and growth of palladium nanoparticles. J Colloid Interface Sci.

[CR35] Yao X, Zhang Y, Du L, Liu J, Yao J (2015). Review of the applications of microreactors. Renew Sust Energ Rev.

[CR36] Zhang L, Hessel V, Peng J (2018). Liquid-liquid extraction for the separation of co(II) from Ni(II) with Cyanex 272 using a pilot scale re-entrance flow microreactor. Chem Eng J.

[CR37] Zhao C-X, He L, Qiao SZ, Middelberg APJ (2011). Nanoparticle synthesis in microreactors. Chem Eng Sci.

