# Supplementary material for: Green method for efficient PdNPs deposition on carbon carrier in the microreactor system
Source: J Nanopart Res. 2018 Sep 7;20(9):239. doi: 10.1007/s11051-018-4337-9 (PMC6132981; doi:10.1007/s11051-018-4337-9)
Supplement: Supplementary file 1 — (DOCX 123 kb) [file 11051_2018_4337_MOESM1_ESM.docx]

**Supplementary materials**

**The continuous synthesis of palladium catalyst in microreactor**

by Magdalena Luty-Błocho*, Marek Wojnicki, Grzegorz Włoch and Krzysztof Fitzner

1. Calculation of reaction time

The value of reaction time (nucleation and autocatalytic growth) was established based on our previous kinetic study[1]. Taking into account, the value of reactor (V_R_ =250µL, Fig. 1) and reaction time (t =2.5s (Fig. 9a) [1], at 40°C) we are able to set the value of flow rate (F_R_) equals 6.0mL/min according to the equation:

$F_{R}=\frac{V_{R}}{t}$

1. Adsorption of ascorbic acid on active carbon surface

In results of mixture 4mL of 0.2mM solution of ascorbic acid with active carbon fibers, the change in spectra were registered. The decrease of maximum value of absorbance confirms, that on the carbon surface, the proces of adsorption of ascorbic acid may take place (Fig. 1S).





Figure 1S. Spectra evolution for an aqueous solution of ascorbic acid being in contact with active carbon fibers. Conditions: C_0, AA_=0.2mM; T=20°C; Optical path length 1cm.

1. Wojnicki, M., K. Fitzner, and M. Luty-Błocho, *Kinetic studies of nucleation and growth of palladium nanoparticles.* Journal of Colloid and Interface Science, 2016. **465**: p. 190-199.
